# Physisorption-assistant optoelectronic synaptic transistors based on Ta_2_NiSe_5_/SnS_2_ heterojunction from ultraviolet to near-infrared

**DOI:** 10.1038/s41377-025-01792-3

**Published:** 2025-03-17

**Authors:** Fan Tan, Chunlu Chang, Nan Zhang, Junru An, Mingxiu Liu, Xingyu Zhao, Mengqi Che, Zhilin Liu, Yaru Shi, Yahui Li, Yanze Feng, Chao Lin, Yuquan Zheng, Dabing Li, Mario Lanza, Shaojuan Li

**Affiliations:** 1 State Key Laboratory of Luminescence Science and Technology, Changchun Institute of Optics, Fine Mechanics and Physics, Chinese Academy of Sciences, Changchun, 130033 China; 2 University of Chinese Academy of Sciences (UCAS), Beijing, 100049 China; 3 State Key Laboratory of Applied Optics, Changchun Institute of Optics, Fine Mechanics and Physics, Chinese Academy of Sciences, Changchun, 130033 China; 4 Key Laboratory of Optical System Advanced Manufacturing Technology, Chinese Academy of Sciences, Changchun, 130033 China; 5 Materials Science and Engineering Program, Physical Sciences and Engineering Division, King Abdullah University of Science and Technology (KAUST), Thuwal, 23955 Saudi Arabia

**Keywords:** Photonic devices, Optical sensors

## Abstract

Neuromorphic computing vision is the most promising technological solution to overcome the arithmetic bottleneck in machine vision applications. All-in-one neuromorphic sensors have been attracting increased attention because they can integrate visual perception, processing, and memory functionalities into one single device. However, the limited responsivity and data retention time of all-in-one neuromorphic sensors usually hinder their potential in multispectral machine vision, especially in the near-infrared (NIR) band which contains critical information for pattern recognition. Here, we demonstrate physisorption-assistant optoelectronic synaptic transistors based on Ta_2_NiSe_5_/SnS_2_ heterojunction, which present tunable synaptic functionality in broadband (375–1310 nm). We propose a strategy about the physisorption-assistant persistent photoconductivity (PAPPC) effect to effectively solve the problem in detecting and storing the NIR light information. Under this strategy, the responsivity and data retention time of our devices were significantly enhanced and prolonged in broadband from 375 to 1310 nm. Further, the devices realize multilevel non-volatile optoelectronic memory through the modulation of several optical and back-gate signals to simulate emotion-controlled learning and memory processes, optical writing-electric erasing, and associative learning. Moreover, we developed a simplified human visual system to simulate color-cognitive perception and memory functions. Our approach offers a route for creating advanced all-in-one neuromorphic sensors and developing neuromorphic computing vision.

## Graphical abstract

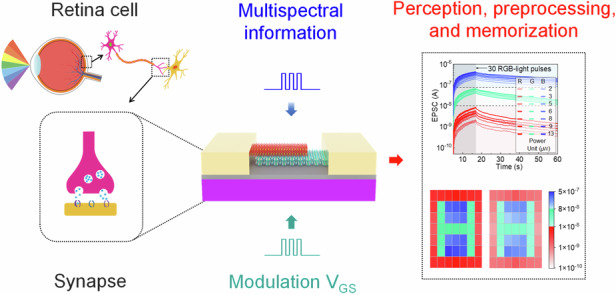

## Introduction

Research in neuroscience and cognitive psychology has shown that the most essential way to acquire information about the outside world is visual perception^1^. Moreover, visual perception is a key technology for security surveillance, autonomous driving, face recognition, photoelectric tracking, and biomedical imaging. However, these application scenarios pose a significant challenge to the arithmetic power of visual perception. The traditional vision sensors utilize the von Neumann architecture, which presents challenges in boosting arithmetic capabilities and reducing power consumption due to the separation of the storage units and the computation units^2,3^. Fortunately, neuromorphic vision sensors offer a highly prospective solution for overcoming the arithmetic bottleneck in visual perception^2–11^. Neuromorphic vision computing dramatically breaks the arithmetic bottleneck by simplifying the raw data and optimizing data transfer between photodetectors and computation units^12^. More importantly, neuromorphic vision sensors hold substantial potential for mimicking the environment adaptation and in-memory sensing capability of human vision system^13^.

To improve information processing efficiency, reduce power consumption, and simplify the design in artificial systems, a highly compact all-in-one neuromorphic sensor has attracted increased attention because it can integrate visual perception, processing, and memory functionalities into one device^14–16^. Recently, the optoelectronic synaptic transistor has been attracting increasing attention as an all-in-one neuromorphic sensor because its three-terminal architecture can receive and read stimuli concurrently, accelerating signal processing^17^. Moreover, the gate electrode, as an additional third terminal, can effectively modulate the synaptic plasticity by regulating the conductance of the channel. The currently reported photoelectric synaptic transistors have demonstrated the in-memory sensing capability, but most of them can only operate at a specific wavelength or limited wavelength range^1,14,16^. This is because the photon energy decreases with the wavelength increasing, and the photoelectric conversion efficiency also decreases rapidly, resulting in the realization of non-volatile storage is also more difficult. A proposed solution involves employing the photogating effect to increase photoelectric conversion efficiency, especially for photons with longer wavelengths. The excellent photoelectric conversion efficiency is attributed to the remarkable photodetection gain: $G=\frac{\tau }{{t}_{{\rm{L}}}}$, where *τ* and *t*_L_ are the lifetime and the transit time of the photocarrier, respectively^18^. Increasing carrier lifetime can increase both photodetection gain and data retention time, which partly solves the problem that optoelectronic synapses can no longer function under longer wavelength light stimulation.

The conventional method to increase the carrier lifetime is to block one type of carrier at the heterojunction interface to prolong the lifetime of another type of carrier, but this is far from enough. Another effective strategy is to prolong the lifetime further by taking advantage of the localized states caused by gas molecules’ physisorption in the air^19^. SnS_2_ is a two-dimensional material widely used for gas detection, due to its abundant intrinsic sulfur vacancies that can be used as gas adsorption sites and extend the carrier lifetime^20^. Besides, the intrinsic defects can effectively improve the performance of optoelectronic synapses by capturing and confining photogenerated carriers^21,22^. To further enhance the performance in NIR light, we use Ta_2_NiSe_5_ as an NIR absorber to improve the NIR adsorption of optoelectronic synaptic transistors. As a ternary chalcogenide with great potential in infrared detection, Ta_2_NiSe_5_ exhibits a direct narrow-band structure with 0.33 eV bandgap, which leads to an excellent NIR absorbance^23^. Accordingly, we use Ta_2_NiSe_5_ and SnS_2_ to form heterojunctions to explore the application of photonic synapses from ultraviolet (UV) to NIR.

In this work, we report physisorption-assistant Ta_2_NiSe_5_/SnS_2_ optoelectronic synaptic transistors which present in-memory sensing capability from UV to NIR regions due to significant PAPPC effect. We propose a strategy to greatly enhance the persistent photoconductivity (PPC) through adsorbing surface states and energy band engineering. Surprisingly, the Ta_2_NiSe_5_/SnS_2_ transistor exhibits not only an outstanding specific detectivity of 4.1 × 10^14^ Jones and an ultra-high external quantum efficiency of 1.7 × 10^6^% but also a superior photoresponsivity of 5.6 × 10^3^ A W^−1^ under 405 nm laser irradiation. Moreover, the device demonstrates a detectivity of 1.1 × 10^12^ Jones and a photoresponsivity of 14.4 A W^−1^ in the NIR spectrum as well. Besides, the device can emulate diverse typical synaptic functions from 375 to 1310 nm. These functions are excitatory post-synaptic current (EPSC), the transitions from short-term plasticity (STP) to long-term plasticity (LTP), pair-pulse facilitation (PPF), emotion-controlled learning and memory process, learning behavior, and frequency-dependent learning properties. Furthermore, the classical Pavlovian associative learning is also simulated successfully, suggesting that our device can handle multiple input signals. Moreover, a proposed neuromorphic multi-spectral human visual system based on the Ta_2_NiSe_5_/SnS_2_ heterojunction mimics the color-cognitive perception and memory capabilities. Our strategy offers a viable approach for designing cutting-edge all-in-one neuromorphic sensors and developing multispectral neuromorphic computing vision. In addition, the applicability of optoelectronic synaptic transistors could be extended to multimodal (vision and olfactory) neuromorphic sensors for precise object recognition by employing the physisorption-assistant approaches.

## Results

### Fabrication and characterization of Ta_2_NiSe_5_/SnS_2_ heterojunction

Figure S1a displayed an optical microscope image of the Ta_2_NiSe_5_/SnS_2_ device. The layered Ta_2_NiSe_5_ and SnS_2_ flakes were obtained from their bulk materials through mechanical exfoliation and titanium/gold (20/80 nm) electrodes were made through ultraviolet photolithography and thermal evaporation. A detailed fabrication process of the Ta_2_NiSe_5_/SnS_2_ heterojunction can be found in the “Device fabrication” section. Next, Raman spectroscopy was employed to investigate phonon vibrations and interlayer coupling within Ta_2_NiSe_5_/SnS_2_ heterojunction, as shown in Fig. S1b. For pure Ta_2_NiSe_5_, there are seven distinct Raman peaks located at 95.7, 121.4, 147.8, 174, 189.4, 213.9, and 287.6 cm^−^^1^, which are related to the *A*_g_ vibration mode (blue line)^24^. For pure SnS_2_ flake, there is only one main peak located at 314.7 cm^−1^, which is associated with *A*_1g_ vibration mode (yellow line)^25^. The Raman modes of both Ta_2_NiSe_5_ and SnS_2_ are observed in the spectra of the Ta_2_NiSe_5_/SnS_2_ overlapped region (red line), manifesting that the Ta_2_NiSe_5_/SnS_2_ heterojunction was effectively fabricated. Subsequently, atomic force microscopy (AFM) was employed to study the thickness and uniformity of the Ta_2_NiSe_5_/SnS_2_ heterojunction, as shown in Fig. S1c. Based on the AFM profiles depicted in Fig. S1d, the thicknesses of Ta_2_NiSe_5_ and SnS_2_ are 39.1 and 8.4 nm, indicating that the two materials are highly uniform. Last, we investigated the surface charge distribution of Ta_2_NiSe_5_ and SnS_2_ by Kelvin probe force microscopy (KPFM) measurement, as depicted in Fig. S1e, f. The KPFM measurements indicated that the Fermi level (*E*_F_) of SnS_2_ is 179.2 mV lower than that of Ta_2_NiSe_5_, which suggests a strong built-in electric field is formed.

### Photoelectric characteristics of Ta_2_NiSe_5_/SnS_2_ device

**Fig. 1:**
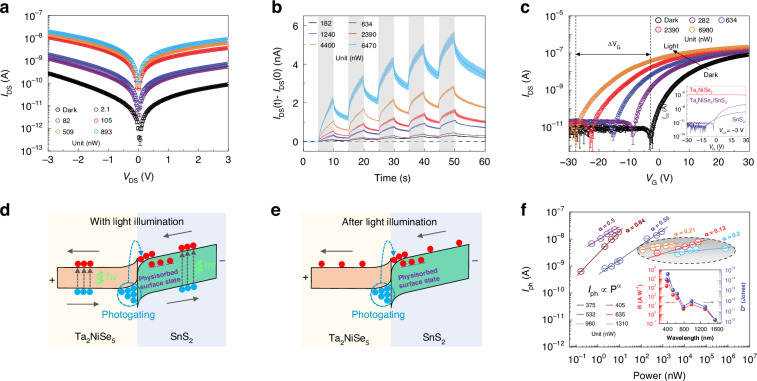
Optoelectronic characterization of the Ta2NiSe5/SnS2 device. **a** Output curves (*I*_DS_–*V*_DS_) in the dark and under illumination with different powers. The incident light is at 532 nm wavelength and *V*_G_ = 0 V. **b** Time-resolved photoresponse of the Ta_2_NiSe_5_/SnS_2_ device at *V*_DS_ = −3 V. The incident light is at 532 nm wavelength and *V*_G_ = 0 V. The gray part represents the light pulses applied to Ta_2_NiSe_5_/SnS_2_ device. **c** Transfer curves (*I*_DS_–*V*_G_) in dark and under illumination with different power. The incident light is at 532 nm wavelength and *V*_DS_ = −1 V. **d** Band diagrams of the junction under light illumination. **e** Band diagrams of the junction after light illumination. **f** Photocurrents at *V*_DS_ = −3 V under lasers from 375 to 1310 nm with various light intensities. All test results are from four parallel tests, data points and plot lines are the mean of the four results, error bars and error bands represent the standard deviation of the four results

To investigate the photoelectric characteristics of the Ta_2_NiSe_5_/SnS_2_ devices under constant light, the source-drain *I*–*V* curves were obtained at various laser powers 532 nm laser, as depicted in Fig. 1a. A clear distinction was observed in *I*–*V* curves with various laser powers, which indicated a strong photo-response. Then, the specific detectivity, external quantum efficiency, and responsivity of the Ta_2_NiSe_5_/SnS_2_ transistors were calculated at *V*_DS_ = −3 V, as shown in Fig. S2. All the performance metrics decrease as the laser power increases, which presents trap-associated photoelectric performance^26^. The highest value of specific detectivity (D* = 2.50 × 10^14^ Jones), external quantum efficiency (EQE = 38810%), and responsivity (*R* = 166.3 A W^−1^) of the Ta_2_NiSe_5_/SnS_2_ device were obtained at the incident power of 2.1 nW, which presents excellent photoelectric characteristics under constant light. To study the transient photoelectricity response of the device, we measured the response of the Ta_2_NiSe_5_/SnS_2_ device under optical pulses of different powers of the 532 nm laser at the condition of *V*_DS_ = −3 V. Figure 1b depicts that the photocurrent of Ta_2_NiSe_5_/SnS_2_ device did not drop immediately to initial current level but still maintain consistent for a specific duration after light excitation, which can be attributed to PPC effect^27^. Furthermore, we found that the decay process can be modulated by applying multiple light pulses, which offers the potential for diverse forms of synaptic plasticity^5,28^. A similar phenomenon can be observed in the broadband from UV to NIR range, as shown in Fig. S3. The statistical robustness of the photoresponse was discussed in Supplementary Note 1, which is crucial for the application of neuromorphic machine vision. The four parallel tests of the three devices exhibit comparable responsivity, thereby confirming the reproducibility of responsivity data. Thus, our devices have great potential for realizing wide-band optoelectronic synaptic applications.

Next, we studied the transfer curves of the Ta_2_NiSe_5_/SnS_2_ transistor to explore the mechanism of PPC generation. Firstly, we examined the transfer characteristic of the Ta_2_NiSe_5_/SnS_2_ transistor in the non-illuminated state and compared it with the transfer characteristic of the pure Ta_2_NiSe_5_ transistor and the pure SnS_2_ transistor, as depicted in the inset of Fig. 1c. The characterization and fabrication details of pure Ta_2_NiSe_5_ and pure SnS_2_ devices can be seen in Fig. S6 and the “Device fabrication” section. The thickness of the pure Ta_2_NiSe_5_ device and pure SnS_2_ device are 30 and 13 nm respectively, which is basically consistent with the two materials in the Ta_2_NiSe_5_/SnS_2_ device. In the inset of Fig. 1c, when *V*_G_ changes within the range of -30 V to 30 V, the current of Ta_2_NiSe_5_ material remains at the level of 10^-5^, indicating that the impact of gate voltage on the carrier density of Ta_2_NiSe_5_ material is negligible. However, the current of the SnS_2_ device changes drastically from 10^-12^ to 10^-9^, spanning 3 orders of magnitude, which shows that the gate voltage can significantly change the carrier density of the SnS_2_ material and the majority of carriers of the SnS_2_ material are electrons. Interestingly, the changing trend of the transfer curve of the Ta_2_NiSe_5_/SnS_2_ device is consistent with that of the SnS_2_ device, which shows that the gate voltage primarily changes the electron density in the channel of the Ta_2_NiSe_5_/SnS_2_ device. It is noted that the current dynamic range of the Ta_2_NiSe_5_/SnS_2_ device is almost two orders of magnitude larger than the SnS_2_ device, which proves that our device exhibits excellent gate control performance. Secondly, we studied the effect of light on the transfer characteristic of the Ta_2_NiSe_5_/SnS_2_ device by irradiating the device with 532 nm light of different powers, as shown in Fig. 1c. The transfer curves of the Ta_2_NiSe_5_/SnS_2_ device gradually shifted to the left and the threshold voltage changes significantly with increasing light power, which suggests that the main component of the photocurrent was photogenerated electrons while the majority of photogenerated holes trapped in the heterojunction^18,29,30^. This phenomenon suggests an asymmetric transport behavior of photogenerated carriers in the device, possibly causing the generation of the PPC.

Accordingly, we illustrate this PPC phenomenon due to the asymmetric transport of photogenerated carriers. According to currently reported studies of two materials (Ta_2_NiSe_5_^23,26,31^, SnS_2_^25,32,33^) and the KPFM measurements (Fig. S1e, f), a type II heterojunction is formed as shown in Fig. S7. At reverse bias (*V*_DS_ = −3 V), the energy band structure of the Ta_2_NiSe_5_/SnS_2_ device will tilt, causing electrons to flow from SnS_2_ to Ta_2_NiSe_5_ and holes to flow in the opposite direction, as shown in Fig. 1d. With light illumination, the photogenerated carriers generated by SnS_2_ are transported to the electrodes under the electric field. However, among photogenerated carriers generated by Ta_2_NiSe_5_, only photogenerated electrons can be transported, while photogenerated holes will be blocked by the interfacial potential barrier. The trapped photogenerated holes create a photogating effect, causing the electrons to undergo multiple cycles in the heterojunction channel, resulting in a significant gain. After light illumination, the blocked photogenerated holes can still induce numerous nonequilibrium electrons to generate a continuously decaying current, as shown in Fig. 1e. Moreover, O_2_ and H_2_O molecules will be absorbed on the surface of the channel of SnS_2_ in the Ta_2_NiSe_5_/SnS_2_ device. The sites of the O_2_ and H_2_O molecules may induce additional localized states to prolong the lifetime of the photogenerated electrons^34,35^, which extremely enhances the PPC phenomenon, as shown in Fig. 1d, e. To verify the role of physisorption assistance in PPC, we compared the time-resolved photoresponse in air and vacuum, as shown in Fig. S8a, b. Under physisorption assistance, the PPC is effectively enhanced and the relaxation time of the decay process (determine the memory time) was boosted by an order of magnitude, which is consistent with our theoretical explanation. It is noted that the photoelectric detection performance (except for the response speed) was almost unaffected by physisorption, as shown in Fig. S8c. Since we deliberately block the carriers of Ta_2_NiSe_5_, which can absorb the NIR photons, the energy band structure of the heterojunction can produce a significant PAPPC effect even under NIR illumination, thus broadening synaptic function to the NIR band.

Subsequently, we investigated the photocurrent of the device under illumination from UV to NIR light to validate our theoretical explanations, as shown in Fig. S9–11. We summarized the specific detectivity and responsivity of the transistors at 375–1550 nm, in the inset of Fig. 1f. The responsivity and detectivity of our device have two peaks at 405 and 980 nm. The peak responsivity reaches 5.6 × 10^3^ A W^−1^ (14.4 A W^−^^1^) and the peak detectivity reaches 4.1 × 10^14^ Jones (1.1 × 10^12^ Jones) at 405 nm (980 nm). The reasonable explanation is that the SnS_2_ (Ta_2_NiSe_5_) materials have an absorption peak at 405 nm (980 nm), which is consistent with previous research reports^21,36^. In addition, by employing a power-law relationship to fit the power-dependent photocurrents, *I*_ph_∝*P*^*α*^ (Fig. 1f), the *α* in the longer wavelength lights (635, 980, and 1550 nm) is significantly inferior to those in the shorter wavelength lights (375, 405, and 532 nm). In the previous studies, the *α* represents the loss in photocarrier transport^37,38^. The lesser the value of *α*, the larger the loss of photocarriers in transport. Considering the cutoff wavelength of SnS_2_ (*λ*_c_ = 563 nm), the longer wavelength lights (635, 980, and 1550 nm) represent the photocurrent generated by Ta_2_NiSe_5_. The low *α* index indicates there exists a massive carrier loss in photocarriers generated by Ta_2_NiSe_5_, which is associated with asymmetric transport of photogenerated carriers that the vast majority of photogenerated holes from Ta_2_NiSe_5_ are blocked by interfacial potential barriers.

### Light tunable synaptic behaviors in Ta_2_NiSe_5_/SnS_2_ device

**Fig. 2:**
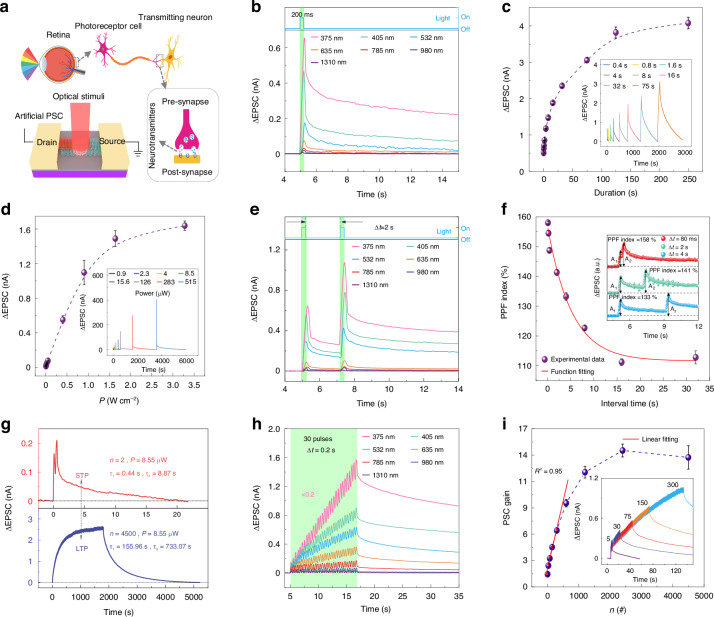
**Light tunable synaptic behaviors in Ta**_**2**_**NiSe**_**5**_**/SnS**_**2**_ **device. a** Schematic of the entire biological visual system. **b** An EPSC of Ta_2_NiSe_5_/SnS_2_ device triggered by an optical pulse. The duration of the optical pulses is 200 ms. The power of the optical pulses from 375 to 1310 nm are 0.15 μW, 0.36 μW, 0.26 μW, 1.89 μW, 5.66 μW, 21.05 μW, and 1.19 mW. **c** Dependence of the EPSC triggered by an optical pulse on the pulse duration. The duration and power of the optical pulses are 200 ms and 13.26 μW. Error bars represent the standard deviation obtained from four times independent tests of Ta_2_NiSe_5_/SnS_2_ device. **d** Dependence of the EPSC triggered by an optical pulse on the power of the pulses. The duration of the optical pulses is 200 ms. Error bars represent the standard deviation obtained from four times independent tests of Ta_2_NiSe_5_/SnS_2_ device. **e** EPSC of Ta_2_NiSe_5_/SnS_2_ device triggered by a pair of optical pulses with a duration of 200 ms. The interval time(△t) between the two pulses is 2 s. The power of the optical pulses from 375 to 1310 nm are 0.15 μW, 0.79 μW, 1.73 μW, 13.26 μW, 6.19 μW, 421 μW, and 1.19 mW. **f** Dependence of the PPF index (defined as *A*_2_/*A*_1_) on △*t*. The red solid line results from the fitting with an exponential function. **g** The dependence of transition from STP to LTP on the quantity of the optical pulses. The duration and interval time of the optical pulses are 200 and 200 ms, respectively. **h** EPSC of Ta_2_NiSe_5_/SnS_2_ device triggered by 30 pulses. The time interval between the pulses is 200 ms. The power of the optical pulses from 375 to 1310 nm are 0.15 μW, 0.02 μW, 0.51 μW, 2.3 μW, 43.04 μW, 219 μW, and 857.77 μW. **i** Dependence of PSC gain on the quantity of the optical pulses. Error bars represent the standard deviation obtained from four times independent tests

Figure 2a illustrates a model of the human visual system. When light enters the eye, light signals are transformed into electrical signals and transmit preprocessed electrical signals into the cerebral cortex by transmitting neurons to visual recognition and perception. When a retinal cell receives a light pulse signal input, the information is stored as an excitatory pulse in the form of exponential decay in post-synaptic current (PSC). This means that all information decays naturally, and only the information that is frequently used is retained, making this a breakthrough in the bottleneck of the von Neumann computing system. Inspired by the retina, the Ta_2_NiSe_5_/SnS_2_ device can perform the neuromorphic function of the photoreceptor cell.

To explore the device’s capability in detecting and recording optical signals, we irradiated the device with a single light pulse of 375–1310 nm with a duration of 200 ms. Figure 2b illustrates the typical EPSC response observed in the Ta_2_NiSe_5_/SnS_2_ device. After light stimuli, the Ta_2_NiSe_5_/SnS_2_ device responds to light signals of various wavelengths with distinct EPSC values and memory time. Therefore, our device has the potential to perceive and distinguish the optical signals from 375 to 1310 nm. More details about the EPSC in NIR can be seen in Fig. S12a. The decrease in EPSC peak value was observed to be significantly correlated with the increase in wavelength, attributed to the weakening of the photogate effect, which will be discussed in Supplementary Note 2. Taking the synaptic behavior at 635 nm as an example, we next discuss the modulation of EPSC by changing light pulse power and duration. In Fig. 2c, as the pulse duration increases, the EPSC peak value also increases, reaching a saturation value of 4.08 nA when the pulse duration is 250 s. This phenomenon closely resembles the variability of EPSC in a biological synapse within a neural system^39^. A similar phenomenon can be observed in NIR, as shown in Fig. S12b. As the optical pulse intensity increases, the maximum value of EPSC also increases, as shown in Fig. 2d. We further investigate the multilevel storage capabilities of our synaptic device under the programming operation of single pulses with various light powers in 635 nm, as shown in Fig. S15. The results show that multilevel discernable current states could be written by light pulses with various light powers and the retention time was higher than 50 s. The above experimental results demonstrate that the Ta_2_NiSe_5_/SnS_2_ transistor has the potential to detect and store multiple light information, such as wavelength, power, and duration of light pulses.

Pair-pulse facilitation (PPF) is one of the prominent characteristics of STP, where consecutive presynaptic stimuli lead to an enhancement of the postsynaptic signal; it is essential to receive and process visual information in real-time^40,41^. In Fig. 2e, the EPSC resulting from a pair of optical spikes is illustrated. The spike duration is 200 ms, and the inter-spike interval (△*t*) is 2 s. It is evident that the EPSC evoked by the second optical spike (*A*_2_) exceeds that evoked by the first optical spike (*A*_1_) in the range of 375–1310 nm, demonstrating the well-established phenomenon of PPF. More details about the PPF in NIR can be seen in Fig. S12c. This phenomenon arises from the fact that the photo-generated carriers from the initial optical pulse do not undergo complete recombination upon stimulation by the subsequent optical pulse. Taking the synaptic behavior at 635 nm as an example, we next investigated the ratio *A*_2_ to *A*_1_ (i.e., PPF index) depending on △*t*, as shown in Fig. 2f. The PPF can be quantified by the ratio of the two EPSCs, that is, PPF index = (*A*_2_/*A*_1_) × 100%. The maximum PPF index of 158% is obtained when the △*t* is 80 ms. The *PPF index* exponentially decays as △*t* increases. A similar phenomenon can be observed in NIR, as shown in Fig. S12d, e. Such observation is consistent with the information process in a biological synapse, and the high *PPF index* is crucial for high-precision decoding and processing of visual information^5^. The above experimental results demonstrate that the Ta_2_NiSe_5_/SnS_2_ device has the potential to preprocess the multispectral information. A detailed comparison between Ta_2_NiSe_5_/SnS_2_ devices and other optoelectronic synaptic devices is presented in Table S1. Our devices possess a distinct advantage in the combined performance of operating wavelength range, PPF index, and power consumption.

In biological systems, the transition from short-term potentiation (STP) to long-term potentiation (LTP) can be induced through repetitive pulsed stimulation, leading to a continuous enhancement of synaptic strength^42^; it is essential for biological memory and learning. Figure 2g shows the transition from STP to LTP by changing the number of light pulses. It is noted that as the number of optical pulses increases from 2 to 4500, the peak value of the EPSC increases dramatically from 0.21 to 2.6 nA and the retention time (i.e., the time when the EPSC peak decays from 90% to 10% of its initial value) increases from 11.29 to 1299.51 s, which indicates that the effectiveness of learning and memory time can be modulated by learning times. To further investigate the transition from STP to LTP, we fit the decay process of EPSC with the double-exponential decay equation:

$$I={I}_{0}+{C}_{1}\exp \left(-\frac{x-{x}_{0}}{{\tau }_{1}}\right)+{C}_{2}\exp \left(-\frac{x-{x}_{0}}{{\tau }_{2}}\right)$$

where relaxation time constant *τ*_1_ and *τ*_2_ correspond to two decay mechanisms with different rates: fast band-to-band transition (*τ*_1_) and slow carrier de-trapping (*τ*_2_), of which the slow process with relaxation time constant *τ*_2_ plays the decisive role in determining the forgetting rate^18,40^. The decay process can be well-fitted by using the double exponential decay function, as shown in Fig. S16. The time constants for the decay of STP and LTP in the Ta_2_NiSe_5_/SnS_2_ device are 8.87 and 733.07 s, respectively (Fig. 2g). This suggests that the rate of forgetting can be significantly modulated by repetitive learning. Subsequently, we investigated the transition from STP to LTP under optical stimulation from 375 to 1310 nm. Figure 2h depicts the EPSC triggered by 30 optical pulses of 375-1310 nm with a duration of 200 ms, demonstrating the emulation of the typical transition from STP to LTP of retinal cells during repeated light stimulations. The time interval (△*t*) between these two optical spikes is 200 ms. More details about the transition from STP to LTP in NIR can be seen in Fig. S12f. The value of EPSC increased significantly with the increase of pulse number during 30 consecutive light pulses, as shown in Fig. 2h. This is attributed to the PAPPC effect due to the large number of photogenerated carriers produced after repeated stimulation with light pulses. The above experimental results demonstrate that the Ta_2_NiSe_5_/SnS_2_ device has the potential to learn and remember multispectral information.

We further studied the key parameters of the LTP which have a major impact on the accuracy of hardware artificial neural networks^43^. Firstly, we investigated the dependence of the EPSC gain (determined as *A*_n_/*A*_1_) on the number of optical spikes which is related to the contrast in neuromorphic imaging and preprocessing^28^, as shown in Fig. 2i. When the pulse number is less than 300, the EPSC gain increases linearly with the pulse number; when the number of pulses is greater than 300, the dependence of the EPSC gain on the number of pulses deviates from linearity and eventually saturates. Next, we studied the dynamic range of the Ta_2_NiSe_5_/SnS_2_ device, as shown in Fig. S17a. The dynamic range is defined by the following equation:

$${{\rm{dynamic}}\; {\rm{range}}}=20\log ({\rm{PSC}}_{\max }/{\rm{PSC}}_{\min })$$

where PSC_max_ is steady state PSC after 30_th_ pulse, and PSC_min_ is steady PSC after 1_st_ pulse^1^. After 30 light pulses, the device exhibits a dynamic range of 10.43 dB, which indicates the potential to contain multiple conductance states and is crucial for high-precision neuromorphic computing^44^. The synaptic performance of the Ta_2_NiSe_5_/SnS_2_ device can be further adjusted to manipulate the PAPPC effect by modifying the gas environment, which was discussed in Supplementary Note 3. Subsequently, we investigated the non-linearity of the Ta_2_NiSe_5_/SnS_2_ device, as shown in Fig. S17b. The formula provided below is utilized to model the LTP process of the synapse^45^:

$${G}_{{\rm{LTP}}}=\frac{B\left(1-{e}^{-\frac{P}{A}}\right)}{1-\,{e}^{-\frac{{P}_{\max }}{A}}}+{G}_{\min }$$

where *G*_LTP_ and *G*_min_ denote the current level under the light pulse and the initial state, respectively. *P* and *P*_max_ represent the pulse number and the maximum number, while *B* and *A* are constants that require to be fitted. *A* is influenced by the nonlinearity of weight update and can be either positive or negative. *B* is a function of *A* within the range of *G*_min_ and *P*. Generally, as *A* approaches 0, the curve’s linearity improves^28^. The nonlinearity of our device, measured at 26.57, is a critical factor for its application in neuromorphic computing. The above experimental results demonstrate that the Ta_2_NiSe_5_/SnS_2_ device has the potential in hardware artificial neural networks.

### The tunable long-term plasticity of the optoelectronic synaptic transistors

**Fig. 3:**
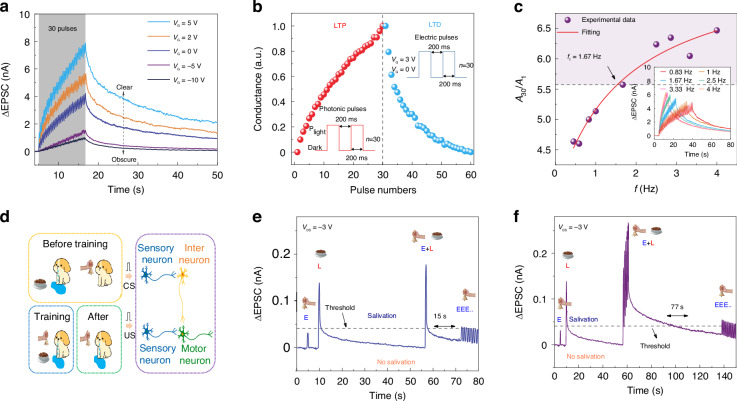
The optoelectronic tunability of long-term plasticity. **a** Dependence of the EPSC triggered by 30 optical pulses on various back-gate voltages. The bias voltage (*V*_DS_), wavelength, duration, and power of the optical pulses are −3 V, 635 nm, 200 ms, and 13.26 μW. **b** LTP/LTD characteristics. Light and voltage pulses for LTP and LTD are depicted in the figures. Photonic pulses conditions of the Ta_2_NiSe_5_/SnS_2_ device are 635 nm, 13.26 μW power, 200 ms width. **c** Dependence of the gain of EPSC (*A*_30_/*A*_1_) on the pulse frequency. The wavelength, duration, and power of the optical pulses are 635 nm, 200 ms, and 13.26 μW. **d** Schematic illustration of Pavlov’s dog experiment and the neural circuit of associative learning. Classical Pavlovian conditioning under **e** one and **f** five training (E + L)

It is widely accepted that moods can influence the processes of learning and forgetting^46^. Because the gate electrode can effectively regulate the conductance of the channel, the Ta_2_NiSe_5_/SnS_2_ device has great potential for simulating the effects of emotion on learning. We simulated the learning progress with 30 continuous light pulses and simulated the forgetting progress with the decay of EPSC after 30 continuous light pulses. Figure 3a shows the EPSC triggered by 30 optical pulses under various gate voltages at 635 nm laser. As the gate voltage varies from −10 to 5 V, the maximum value of the EPSC increases as the gate voltage increases. We can define the person has a neutral mood when *V*_g_ = 0 V. For *V*_g_ < 0 V and *V*_g_ > 0 V, the person has a negative mood and a positive mood, respectively. It is noted that the learning results are forgotten more quickly (slowly) for a negative (positive) mood, which is consistent with the mood’s effect on human learning.

It has been proposed that long-term potentiation (LTP) and long-term depression (LTD) are crucial for the memories encoding^47^. LTP and LTD of the Ta_2_NiSe_5_/SnS_2_ device are updated by optical and electrical pulses, respectively, as shown in Fig. 3b. The LTP and LTD characteristics of the Ta_2_NiSe_5_/SnS_2_ device depend on the pulse number of light pulses and gate-voltage pulses, separately. Figure S21a illustrates the use of light spikes and gate-voltage pulses (*V*_g_ = 3 V) to control synaptic function, transitioning from LTP to LTD. This involves potentiation after 30 consecutive optical stimuli, followed by depression induced by 30 gate-voltage pulses. After 30 optical pulses, the current increases significantly due to the accumulation of photogenerated holes, as shown in Fig. S21b. When positive gate voltage pulses are applied to the Ta_2_NiSe_5_/SnS_2_ device, the interfacial barrier of the heterojunction decreases, and the blocked photogenerated holes are released under the electric field, as shown in Fig. S21c. After 30 gate voltage pulses, the Ta_2_NiSe_5_/SnS_2_ device current returns to its initial value due to the release of photogenerated holes, as shown in Fig. S21d.

LTP of the Ta_2_NiSe_5_/SnS_2_ device can be modulated by the frequency (*f*) of the optical pulses, as shown in Fig. 3c. The frequency of the optical pulses (*f*) is defined by the following equation: *f* = *t*_2_/(*t*_1_ + *t*_2_), where *t*_1_ is the duration of light pulses, *t*_2_ is the interval of light pulses. The gain of the EPSC after 30 optical pulses (*A*_30_/*A*_1_) increases with the increase of the pulse frequency, which presents a high-pass filtering characteristic. The high-pass filter is able to effectively block signals with a low frequency below the cut-off value while permitting high-frequency signals to pass through, which is essential for preprocessing images in the human retina^48^. To further investigate the cutoff frequency of our device, we fit the frequency-dependence of EPSC gain with the sigmoidal function: $g(f)=({a}_{1}-{a}_{2})/(1+{(f/{f}_{c})}^{p})+{a}_{2}$, where *p* is the order of the function, *f*_*c*_ is the cut-off frequency, *a*_1_ and *a*_2_ are the initial and final amplitude^48,49^. The gain curve can be best fitted with *p* = 1. The fitting results in a value of 1.67 Hz for *f*_*c*_. The similarity to the high-pass filtering characteristic of biological synapses indicates that the Ta_2_NiSe_5_/SnS_2_ device holds promise as a high-pass filter for image preprocessing, including image sharpening^48^.

Due to the tunability and long-term plasticity of the optoelectronic synaptic transistors, the Ta_2_NiSe_5_/SnS_2_ device has the potential to simulate associative learning between two physical inputs, such as the Pavlovian conditioning, as shown in Fig. 3d. The dog will instinctively secrete saliva when it sees food. We call the behavior of secreting saliva as the unconditional reflex (UR) and call the behavior of seeing food as the unconditional stimulus (US). The dog will not secrete saliva when it hears a bell. We call the behavior of hearing a bell as the neutral stimulus (NS). If we deliberately ring the bell while the dog is eating, the dog associates hearing the bell with having food to eat. After training, the dog will associate US with NS and will salivate even if it only hears a bell. We call the behavior of the dog secreting saliva when it hears a bell as the conditional reflex (CR)^5,48^.

To mimic this Pavlovian associative learning, we defined the application of a gate voltage pulse (+0.1 V, 200 ms) as a bell stimulus, the application of a light pulse (13.26 μW, 200 ms) as a food stimulus, and the simultaneous application of both a gate voltage pulse and a light pulse as one training to make the dog believes that there exists a connection between food stimulation and ringing stimulation, as shown in Fig. 3e. The drain current is defined as the terminal that detects the CR and UR output signals. The threshold current (*I*_th_ = 41 pA) for the salivary response was marked with a black dashed line in Fig. 3e, f. When we apply the gate voltage pulse (NS), only a weak current pulse (31.5 pA) is generated in the channel, which does not reach the threshold. That is, applying only a bell stimulus (NS) does not make a puppy salivate (UR). When we applied the light pulse as food stimulation (US), the current rapidly increased to 145 pA, significantly above the threshold, causing the puppy to salivate (UR). However, the current rapidly decreased and fell below the threshold after the light pulse was withdrawn. When we applied both a gate voltage pulse and a light pulse as food and ringing stimuli (US + NS), the current rapidly increased to 177 pA and exceeded the threshold causing the puppy to salivate, suggesting the link between food and ringing was established. After a training session and the current dropped to about 20 pA, if multiple gate voltage pulses are applied again, only the first three gate voltage pulses trigger currents above the threshold, which means that the training can only provisionally associate NS with CS to induce CR. This phenomenon is similar to the biological nervous system. In order to eliminate redundant information from the nervous system, CRs that are not trained regularly will fade away. With repeated training, it is possible to maintain the CR for a longer time. After 5 training sessions and the current dropped to about 20 pA, CR will be strengthened and the triggered current is above the threshold even after 19 gate voltage pulses, as shown in Fig. 3f. Interestingly, even at 980 nm, our device is able to achieve this Pavlovian association learning, as shown in Fig. S22. This suggests that our device can realize the basic function of synapses even in the NIR band.

### Simulation of the behavior of the human visual system

**Fig. 4:**
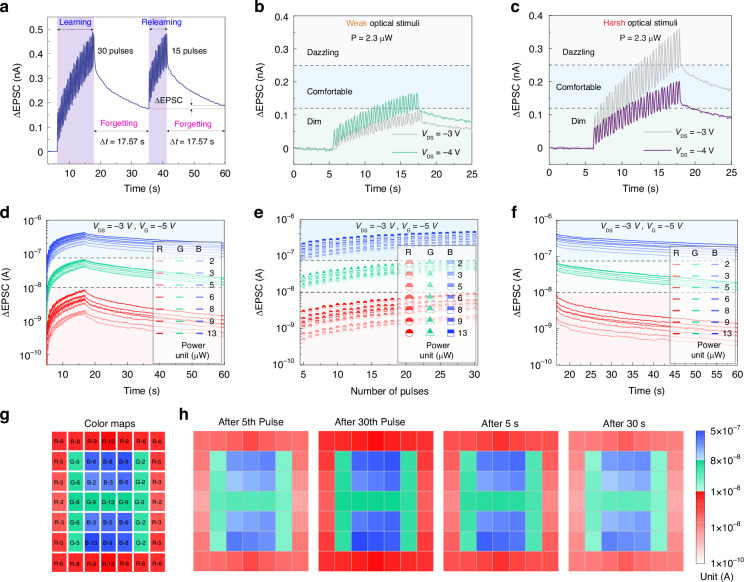
Simulation of the behavior of human visual system. **a** The “learning-forgetting-relearning” process triggered by multiple light pulses. **b**, **c** Simulation of the light adaptive behavior of human visual system in 635 nm wavelength application scenario. **d** EPSC variations of Ta_2_NiSe_5_/SnS_2_ device under RGB-light pulses (635, 532, and 405 nm) with various light power from 2 to 13 μW. RGB pulse conditions: 200 ms width and 200 ms interval. **e** EPSC distribution depending on the number of RGB-light pulses extracted from (**d**). **f** The decay of the EPSC after 30 RGB-light pulses extracted from (**d**). **g** We used the RGB-light information shown in the figure for single-point scanning imaging. For example, “R-6” represents red light pulses (635 nm) with 6 μW. The duration, interval, and quantity of all the optical are 200 ms, 200 ms, and 30, respectively. **h** 2D contour map of PSCs measured from Ta_2_NiSe_5_/SnS_2_ device at different pulses and retention time conditions. All measurements were performed at a drain voltage of −3 V and a gate voltage of −5 V

Usually, humans acquire new knowledge through three processes: initial learning, partial forgetting, and relearning. During relearning, human can recover their memory to the original level with only a small amount of learning and the stability is greatly enhanced^48^. We used light pulses to simulate the process of learning and re-learning and used a dark-state environment to simulate the natural decay process of learning memory (Fig. 4a). During the first learning process, the current increased to 480 pA, then after a 17.57 s forgetting process, the current decreased to 175 pA. During the relearning process, the current recovered to 480 pA with 15 pulses, which indicates a progressive learning efficiency in the relearning process. Similarly, undergoing the same forgetting process, the current only decays to 190 pA, which reflects the ability to make the memory effect significantly stronger through learning and reviewing, which is similar to the Ebbinghaus forgetting curve in brain memory^40^.

A key function in the human visual system is adaptation to light power, which allows the human visual system to process visual information in environments with drastic changes in light power^5^. When exposed to dim (or harsh) light, the human eye will initially feel uncomfortable and progressively become able to see them following visual adaptation. If the light is too dim, the device can amplify the photocurrent by increasing the bias voltage, as shown in Fig. 4b. If the light is too harsh, the device can reduce the background noise by reducing the bias voltage, as shown in Fig. 4c. Hence, the simple-structure Ta_2_NiSe_5_/SnS_2_ device shows promising potential to be employed as an artificial pupil in artificial visual systems.

Next, we explored the potential of the Ta_2_NiSe_5_/SnS_2_ device as a retinal cell for color recognition applications. In the retina, the short, middle, and long wavelength-sensitive cone photoreceptors are responsible for acquiring the visible light information to produce color vision^50^. Inspired by the retina, we used RGB-light pulses (R:635 nm for red color; G: 532 nm for green color; B: 405 nm for blue color) with various light intensities from 2 to 13 μW to illuminate the Ta_2_NiSe_5_/SnS_2_ device to mimic the color recognition behavior of the retinal cell. The numbers of red color, green color, and blue color pulses are all 30. As shown in Fig. 4d, it is noted that the EPSC currents corresponding to the three different colors (red, green, and blue) were distinguished. To further explore the color-discrimination capability, we studied whether there is color overlap for EPSC currents. Specifically, the largest EPSC value of red color should be lower than the least EPSC value of green color and blue color in each state^1,50^. Similarly, the largest EPSC value of green color should be lower than the least EPSC value of blue color in each state. If the above two points are met at the same time, we believe that there is no color overlap in EPSC current. In Fig. 4e, we found that from the 5_th_ to 30_th_ RGB-light pulses, the EPSC of R-light is always lower than that of the G-light and B-light. Similarly, the EPSC of the G-light is always lower than that of the B-light. In Fig. 4f, there is no color overlap in the EPSC currents corresponding to the three different colors after 30 RGB-light pulses with various intensities within 43.2 s. As a result, the Ta_2_NiSe_5_/SnS_2_ device is able to effectively discriminate the RGB light in the range of 213 μW without mixing color. Subsequently, we measured the RGB imaging of the device using single-point scanning.

Accordingly, we performed a neuromorphic imaging experiment by single-point scanning. Specifically, we performed 49 independent repetitions of light stimulation on the Ta_2_NiSe_5_/SnS_2_ device to obtain a 7 × 7-pixel color picture. Each light stimulation consisted of 30 continuous light pulses. Take the first light stimulation as an example, we applied the color information of “R-6” in the first row and first column of the color map (Fig. 4g) to the device in the form of 30 continuous 635 nm light pulses at 6.3 μW. We recorded the PSC change of the Ta_2_NiSe_5_/SnS_2_ device and did not perform the next light stimulation until the PSC returned to its initial value. After 49 independent repetitions of the experiment, we achieve the neuromorphic imaging, as shown in Fig. 4h. We found that the Ta_2_NiSe_5_/SnS_2_ device achieved color discrimination of RGB-light with different intensities after the 5th pulse. As the pulse number increased, the color difference became more obvious, which indicates the ability of color recognition can be strengthened through multiple learning. Besides, we found that the Ta_2_NiSe_5_/SnS_2_ device can accurately remember the color information in the 30 s. Our devices show great potential for the recognition, preprocessing, and memory of color information.

## Discussion

To summarize, we have demonstrated the physisorption-assistant optoelectronic synaptic transistors with a prominent performance in recognition, preprocessing, and memory of multispectral information. We propose an effective strategy to greatly enhance the performance of optoelectronic synaptic transistors through physisorption surface state and energy band engineering. Under this strategy, our device exhibits a high specific detectivity of 4.1 × 10^14^ Jones (1.1 × 10^12^ Jones), an ultra-high external quantum efficiency of 1.7 × 10^6^% (1.8 × 10^3^%), and responsivity of 5.6 × 10^3^ A W^−1^ (14.4 A W^−1^) in visible (NIR) light. Due to the PAPPC effect, our device is capable of emulating typical synaptic functions across a broad spectrum ranging from UV to NIR, including EPSC, PPF, and the conversion from STM to LTM. The synaptic plasticity of the device can be modulated by gate voltage pulses, resulting in distinguishable, multi-level nonvolatile memory-based electronic synaptic behavior, which is eventually able to mimic modulation of emotions on visual learning and memory processes and optical write-electro-erase. Furthermore, with the synergistic action of light and gate voltage, our optoelectronic synaptic transistor endowed the emulation of Pavlov’s associative learning. Moreover, we developed a simplified human visual system to simulate color-cognitive perception and memory functions. Our approach offers a route for creating advanced all-in-one neuromorphic sensors and developing neuromorphic computing vision. In the future, gas adsorption-assisted optoelectronic synaptic devices are expected to handle different types of stimuli and are becoming an important trend in sensing technology aiming at better recognition accuracy and robustness.

## Materials and methods

### Device fabrication

The Ta_2_NiSe_5_ and SnS_2_ flakes were mechanically exfoliated from bulk single crystals purchased from HQ Graphene Ltd and Six Carbon Technology Ltd. by using scotch tape and transferred to Polydimethylsiloxane (PDMS) film (METATEST Corporation, China). The Ta_2_NiSe_5_ flake was transferred as the bottom material of the heterojunction onto the pre-cleaned Si/SiO_2_ (285 nm) substrate by PDMS using a high-precision 2D material transfer platform (METAT-EST Corporation, China, E1-T). Next, SnS_2_ flakes were successfully transferred as the top material of the heterojunction to the Ta_2_NiSe_5_ side using the same method. The metal electrodes with a spacing of 20 μm were pattered by UV-lithography. Then, titanium (Ti)/gold (Au) contacts were deposited with a thickness of 20/80 nm by thermal evaporation. The vdWs heterojunction will be annealed at 200 °C under N_2_ gas for 20 min to ensure closer stacking at the interface. Subsequently, we used the same process to make the pure Ta_2_NiSe_5_ device and the pure SnS_2_ device.

### Characterization of Ta_2_NiSe_5_/SnS_2_ device

The morphology of each material was examined with the use of the Optical Microscope (BX51, OLMPUS). The Raman spectra were acquired using a WITec micro-Raman system (532 nm laser). AFM and KPFM images of the Ta_2_NiSe_5_/SnS_2_ heterojunction were collected by Asylum Research Cypher S (Oxford).

### Optoelectrical measurement

The electrical tests were carried out under typical environmental conditions at standard room temperature. A semiconductor parameter analyzer (Keithley 4200) was employed to characterize the static behaviors of the optoelectronic synaptic transistors. The illumination conditions were provided by different lasers: IR (785, 980, 1310, and 1550 nm) and RGB-light (405, 532, and 635 nm) and ultraviolet (375 nm). The temporal response and synaptic characteristics of the optoelectronic synaptic transistors were characterized by a semiconductor parameter analyzer (METATEST Corporation, China, Metatest E2).

*Synaptic test in gas environment*: The synaptic test in gas environment were carried out in the probe station. The gas sensing performance of the sensor was measured by monitoring the variation of the current (Tektronix, USA, Keithley 2636B) in the air and the target gas. The three mass flow controllers (Seven-star, China, CS-200A) were used to vary the O_2_ amount from 50% to 100% by changing the flow ratio between O_2_ and N_2_.

### Supporting information

Detailed information about characterization of Ta_2_NiSe_5_/SnS_2_ heterojunction, photoelectric characteristics of Ta_2_NiSe_5_/SnS_2_ device, synaptic characteristics of Ta_2_NiSe_5_/SnS_2_ device.

## Supplementary information

Supplementary information for Physisorption-Assistant Optoelectronic Synaptic Transistors Based on Ta2NiSe5/SnS2 Heterojunction from Ultraviolet to Near-Infrared

## Data Availability

The data that support the findings of this study are available from the corresponding author upon reasonable request.

## References

[1] Lee, J. Light-enhanced molecular polarity enabling multispectral color-cognitive memristor for neuromorphic visual system. *Nat. Commun.* **14**, 5775 (2023). 37723149 10.1038/s41467-023-41419-y PMC10507016

[2] Merolla, P. A. et al. A million spiking-neuron integrated circuit with a scalable communication network and interface. *Science* **345**, 668–673 (2014). 25104385 10.1126/science.1254642

[3] Cho, S. W. et al. Progress of materials and devices for neuromorphic vision sensors. *Nano-Micro Lett.* **14**, 203 (2022). 10.1007/s40820-022-00945-y PMC9569410 36242681

[4] Manipatruni, S., Nikonov, D. E. & Young, I. A. Beyond CMOS computing with spin and polarization. *Nat. Phys.* **14**, 338–343 (2018).

[5] Ci, W. J. et al. All‐in‐one optoelectronic neuristor based on full‐vdW two‐terminal ferroelectric p–n heterojunction. *Adv. Funct. Mater.* **34**, 2305822 (2024).

[6] Jiang, T. et al. Retina-inspired organic neuromorphic vision sensor with polarity modulation for decoding light information. *Light* **12**, 264 (2023). 10.1038/s41377-023-01310-3 PMC10628194 37932276

[7] Cheng, Y. et al. Photonic neuromorphic architecture for tens-of-task lifelong learning. *Light* **13**, 56 (2024). 10.1038/s41377-024-01395-4 PMC10894876 38403652

[8] Xie, Z. W. et al. Nonvolatile and reconfigurable two-terminal electro-optic duplex memristor based on III-nitride semiconductors. *Light* **13**, 78 (2024). 10.1038/s41377-024-01422-4 PMC10980680 38553460

[9] Fu, X. et al. Graphene/MoS_2−x_O_x_/graphene photomemristor with tunable non-volatile responsivities for neuromorphic vision processing. *Light* **12**, 39 (2023). 10.1038/s41377-023-01079-5 PMC9905593 36750548

[10] Kumar, D. et al. Artificial visual perception neural system using a solution-processable MoS_2_-based in-memory light sensor. *Light* **12**, 109 (2023). 10.1038/s41377-023-01166-7 PMC10162957 37147334

[11] Yu, S. et al. A von-Neumann-like photonic processor and its application in studying quantum signature of chaos. *Light* **13**, 74 (2024). 10.1038/s41377-024-01413-5 PMC10940704 38485915

[12] Zhou, F. C. & Chai, Y. Near-sensor and in-sensor computing. *Nat. Electron.* **3**, 664–671 (2020).

[13] Renner, A. et al. Neuromorphic visual scene understanding with resonator networks. *Nat. Mach. Intell.* **6**, 641–652 (2024).

[14] Jiang, T. et al. Tetrachromatic vision-inspired neuromorphic sensors with ultraweak ultraviolet detection. *Nat. Commun.* **14**, 2281 (2023). 37085540 10.1038/s41467-023-37973-0 PMC10121588

[15] Jayachandran, D. et al. A low-power biomimetic collision detector based on an in-memory molybdenum disulfide photodetector. *Nat. Electron.* **3**, 646–655 (2020).

[16] Zhu, Q. B. et al. A flexible ultrasensitive optoelectronic sensor array for neuromorphic vision systems. *Nat. Commun.* **12**, 1798 (2021). 33741964 10.1038/s41467-021-22047-w PMC7979753

[17] Shi, J. et al. A correlated nickelate synaptic transistor. *Nat. Commun.* **4**, 2676 (2013). 24177330 10.1038/ncomms3676

[18] Liu, M. X. et al. Photogating-assisted tunneling boosts the responsivity and speed of heterogeneous WSe_2_/Ta_2_NiSe_5_ photodetectors. *Nat. Commun.* **15**, 141 (2024). 38167874 10.1038/s41467-023-44482-7 PMC10762006

[19] Peng, R. X. et al. Programmable graded doping for reconfigurable molybdenum ditelluride devices. *Nat. Electron.* **6**, 852–861 (2023).

[20] Huang, Y. et al. Tin disulfide—an emerging layered metal dichalcogenide semiconductor: materials properties and device characteristics. *ACS Nano* **8**, 10743–10755 (2014). 25247490 10.1021/nn504481r

[21] Zhou, X. et al. Large-size growth of ultrathin SnS_2_ nanosheets and high performance for phototransistors. *Adv. Funct. Mater.* **26**, 4405–4413 (2016).

[22] Cheng, R. Q. et al. High-performance, multifunctional devices based on asymmetric van der Waals heterostructures. *Nat. Electron.* **1**, 356–361 (2018).

[23] Qiao, J. et al. Perovskite quantum Dot‐Ta_2_NiSe_5_ mixed‐dimensional van der Waals heterostructures for high‐performance near‐infrared photodetection. *Adv. Funct. Mater.* **32**, 2110706 (2022).

[24] Yan, J. et al. Strong electron–phonon coupling in the excitonic insulator Ta_2_NiSe_5_. *Inorg. Chem.* **58**, 9036–9042 (2019). 31246443 10.1021/acs.inorgchem.9b00432

[25] Zhou, X. et al. Tunneling diode based on WSe_2_/SnS_2_ heterostructure incorporating high detectivity and responsivity. *Adv. Mater.* **30**, 1703286 (2018). 10.1002/adma.201703286 29315847

[26] Guo, T. T. et al. High-gain MoS_2_/Ta_2_NiSe_5_ heterojunction photodetectors with charge transfer and suppressing dark current. *ACS Appl. Mater. Interfaces* **14**, 56384–56394 (2022). 36484601 10.1021/acsami.2c17495

[27] Sumanth, A. et al. A review on realizing the modern optoelectronic applications through persistent photoconductivity. *J. Phys. D* **55**, 393001 (2022).

[28] Deng, Y. et al. Intrinsic defect‐driven synergistic synaptic heterostructures for gate‐free neuromorphic phototransistors. *Adv. Mater.* **36**, 2309940 (2024). 10.1002/adma.202309940 38373410

[29] Wang, Q. S. et al. Nonvolatile infrared memory in MoS_2_/PbS van der Waals heterostructures. *Sci. Adv.* **4**, eaap7916 (2018). 29770356 10.1126/sciadv.aap7916 PMC5954648

[30] Liu, C. H. et al. Graphene photodetectors with ultra-broadband and high responsivity at room temperature. *Nat. Nanotechnol.* **9**, 273–278 (2014). 24633521 10.1038/nnano.2014.31

[31] Zheng, T. et al. Self-powered photodetector with high efficiency and polarization sensitivity enabled by WSe_2_/Ta_2_NiSe_5_/WSe_2_ van der Waals dual heterojunction. *ACS Appl. Mater. Interfaces* **15**, 29363–29374 (2023). 37294943 10.1021/acsami.3c04147

[32] Gao, F. et al. High‐performance van der Waals metal‐insulator‐semiconductor photodetector optimized with valence band matching. *Adv. Opt. Mater.* **31**, 2104359 (2021).

[33] Zhang, L. et al. Ultrahigh-sensitivity and fast-speed solar-blind ultraviolet photodetector based on a broken-gap van der Waals heterodiode. *ACS Appl. Mater. Interfaces* **15**, 14513–14522 (2023). 10.1021/acsami.2c20546 36913956

[34] Zhao, Y. et al. Light-tunable polarity and erasable physisorption-induced memory effect in vertically stacked InSe/SnS_2_ self-powered photodetector. *ACS Nano* **16**, 17347–17355 (2022). 36153977 10.1021/acsnano.2c08177

[35] Wu, G. et al. Miniaturized spectrometer with intrinsic long-term image memory. *Nat. Commun.* **15**, 676 (2024). 38263315 10.1038/s41467-024-44884-1 PMC10805890

[36] Wang, W. J. et al. Optical anisotropy and polarization selectivity in MoS_2_/Ta_2_NiSe_5_ van der Waals heterostructures. *Appl. Phys. Lett.* **122**, 233101 (2023).

[37] Wang, Y. R. et al. Vertical barrier heterostructures for reliable, robust, and high‐performance ultraviolet detection. *Small* **18**, 2204021 (2022). 10.1002/smll.202204021 36116119

[38] Zhang, Q. Y. et al. Ta_2_NiSe_5_/MoTe_2_/graphene van der Waals heterostructures toward ultrabroadband and polarization‐sensitive imaging. *Adv. Opt. Mater.* **12**, 2302958 (2024).

[39] Yang, Y. & Lisberger, S. G. Purkinje-cell plasticity and cerebellar motor learning are graded by complex-spike duration. *Nature* **510**, 529–532 (2014). 24814344 10.1038/nature13282 PMC4132823

[40] Liu, Y. Q. et al. CuInP_2_S_6_‐based electronic/optoelectronic synapse for artificial visual system application. *Adv. Funct. Mater.* **34**, 2306945 (2024).

[41] Han, C. et al. Light‐stimulated synaptic transistor with high PPF feature for artificial visual perception system application. *Adv. Funct. Mater.* **32**, 2113053 (2022).

[42] Yang, X. X. et al. Mechanoplastic tribotronic floating‐gate neuromorphic transistor. *Adv. Funct. Mater.* **30**, 2002506 (2020).

[43] Fang, P. J. et al. Synaptic properties of plasma-treated SnS_2_/h-BN van der Waals heterostructure. *Appl. Phys. Lett.* **122**, 223101 (2023).

[44] Liu, X. X. et al. An optoelectronic synapse based on two‐dimensional violet phosphorus heterostructure. *Adv. Sci.* **10**, 2301851 (2023). 10.1002/advs.202301851 PMC10401094 37229772

[45] Choi, S. et al. SiGe epitaxial memory for neuromorphic computing with reproducible high performance based on engineered dislocations. *Nat. Mater.* **17**, 335–340 (2018). 29358642 10.1038/s41563-017-0001-5

[46] Xie, C. et al. Ultrasensitive broadband phototransistors based on perovskite/organic-semiconductor vertical heterojunctions. *Light* **6**, e17023 (2017). 10.1038/lsa.2017.23 PMC6062319 30167278

[47] Nabavi, S. et al. Engineering a memory with LTD and LTP. *Nature* **511**, 348–352 (2014). 24896183 10.1038/nature13294 PMC4210354

[48] Zhang, J. Y. et al. Retina‐inspired artificial synapses with ultraviolet to near‐infrared broadband responses for energy‐efficient neuromorphic visual systems. *Adv. Funct. Mater.* **33**, 2302885 (2023).

[49] Mapelli, J., Gandolfi, D. & D’Angelo, E. High-pass filtering and dynamic gain regulation enhance vertical bursts transmission along the mossy fiber pathway of cerebellum. *Front. Cell. Neurosci.* **4**, 14 (2010). 20577586 10.3389/fncel.2010.00014 PMC2889686

[50] Jo, C. et al. Retina‐inspired color‐cognitive learning via chromatically controllable mixed quantum dot synaptic transistor arrays. *Adv. Mater.* **34**, 2108979 (2022). 10.1002/adma.202108979 35044005

