# Supplementary material for: Physisorption-assistant optoelectronic synaptic transistors based on Ta2NiSe5/SnS2 heterojunction from ultraviolet to near-infrared
Source: Light Sci Appl. 2025 Mar 17;14:122. doi: 10.1038/s41377-025-01792-3 (PMC11911395; doi:10.1038/s41377-025-01792-3)
Supplement: Supplementary file 1 — Supplementary information for Physisorption-Assistant Optoelectronic Synaptic Transistors Based on Ta2NiSe5/SnS2 Heterojunction from Ultraviolet to Near-Infrared [file 41377_2025_1792_MOESM1_ESM.docx]

**Supporting Information**

**Physisorbed-assistant Optoelectronic Synaptic Transistors Based on Ta_2_NiSe_5_/SnS_2_ Heterojunction with Ultraviolet to Near-Infrared Broadband**

*Fan Tan,**^1,2^ Chunlu Chang, ^1,2^ Nan Zhang,^1,*^ Junru An, ^1,2^ Mingxiu Liu, ^1,2^ Xingyu Zhao, ^1,2^ Mengqi Che, ^1,2^ Zhilin Liu, ^1,2^ Yaru Shi, ^1,2^ Yahui Li, ^1,2^ Yanze Feng, ^1,2^ Chao Lin,^3,4^ Yuquan Zheng,^3,4^ Dabing Li,^1^ Mario Lanza^5^ and Shaojuan Li^1,*^*

^1^State Key Laboratory of Luminescence Science and Technology, Changchun Institute of Optics, Fine Mechanics and Physics, Chinese Academy of Sciences, Changchun 130033, China

^2^University of Chinese Academy of Sciences (UCAS), Beijing 100049, China

^3^State Key Laboratory of Applied Optics, Changchun Institute of Optics, Fine Mechanics and Physics, Chinese Academy of Sciences, Changchun 130033, China

^4^Key Laboratory of Optical System Advanced Manufacturing Technology, Chinese Academy of Sciences, Changchun 130033, China

^5^Materials Science and Engineering Program, Physical Sciences and Engineering Division, King Abdullah University of Science and Technology (KAUST) Thuwal 23955, Saudi Arabia

*Address correspondence to (N. Zhang) zhangnan@ciomp.ac.cn and (S.J. Li) lishaojuan@ciomp.ac.cn

**Figure S1. Characterization of the Ta_2_NiSe_5_/SnS_2_ heterojunction. a)** Optical microscope images of the Ta_2_NiSe_5_/SnS_2_ heterojunction. **b)** The non-polarized Raman spectra collected from isolated Ta_2_NiSe_5_, SnS_2_, and the overlapped heterojunction region. **c)** AFM topography of the Ta_2_NiSe_5_/SnS_2_ heterojunction. **d)** Thickness measurement taken along the green arrow in **c)**. **e)** Kelvin probe force microscopy (KPFM) image that reveals the surface potential difference of the Ta_2_NiSe_5_/SnS_2_ heterojunction. **f)** Surface potential measurement taken along the green arrow in **e)**.

**Figure S2. a)** Responsivity and photocurrent of the Ta_2_NiSe_5_/SnS_2_ device under 532 nm. **b)** Detectivity and EQE of the Ta_2_NiSe_5_/SnS_2_ device under 532 nm.

**Figure S3. Time-resolved photoresponse of the Ta_2_NiSe_5_/SnS_2_ device under 375 nm to 1550 nm light illuminations.**

**Supplementary Note 1. The statistical robustness of the photoresponse of the Ta_2_NiSe_5_/SnS_2_ device.**

We measured the optical responses of three devices to verify the reproducibility of the device’s responsivity data. In Figure S4a-f, the three devices are fabricated utilizing an identical manufacturing process and possess comparable material thickness. In Figure S4g-i, the conduction direction and photocurrent of these three devices are comparable, with only minor variations in performance. In Figure S4j-l, the three devices exhibit comparable responsivity, thereby confirming the reproducibility of the prototype device's responsivity data. In addition, we did four parallel tests on the output curves (*I*_DS_-*V*_DS_) and time-resolved photoresponse of the Ta_2_NiSe_5_/SnS_2_ device (device 3, as shown in Figure S4c) to validate the data consistency, as shown in Figure S5. In Figure S5a, we measured the steady-state optical responses (*I*_DS_-*V*_DS_) of the device at 532 nm. The results demonstrate that the output curves perfectly coincide, with only a marginal distinction observed in the dark current curves. In Figure S5b, we measured the transient optical responses (*I*_DS_-*T*) of the device at 532 nm. The experimental results demonstrate that the IT curves for the device exhibit remarkable similarity when subjected to illumination from 5 optical pulses at varying laser powers, thereby affirming the device’s reliable transient optical response. In Figure S5c-d, the responsivity and retention time were recorded in these four parallel tests, and the data exhibited minimal fluctuations. The results of four parallel tests demonstrate the consistent and reliable nature of both the steady-state optical response and transient optical response exhibited by the device.

**Figure S4. The consistency of performance across different devices (which is corresponding to device 1,2 and 3). a-c)** Optical image of Ta_2_NiSe_5_/SnS_2_ devices. **d-f)** Thickness measurement of Ta_2_NiSe_5_/SnS_2_ devices. **g-i)** Output curves (*I*_DS_-*V*_DS_) of the Ta_2_NiSe_5_/SnS_2_ devices. **j-l)** Responsivity of Ta_2_NiSe_5_/SnS_2_ devices.

**Figure S5. The uniformity of the same device across parallel tests. a)** The output curves (*I*_DS_-*V*_DS_) of the Ta_2_NiSe_5_/SnS_2_ device from 4 parallel tests. **b)** Time-resolved photoresponse of the Ta_2_NiSe_5_/SnS_2_ device from 4 parallel tests. The duration and interval time of the 5 optical pulses at various laser powers are 5 s. **c)** The responsivity of the Ta_2_NiSe_5_/SnS_2_ device from 4 parallel tests at *V*_DS_ = -3 V. **d)** The retention time of the Ta_2_NiSe_5_/SnS_2_ device from 4 parallel tests at *V*_DS_ = -3 V.

**Figure S6. Electrical characterization of Ta_2_NiSe_5_ FETs, SnS_2_ FETs, and Ta_2_NiSe_5_/SnS_2_ heterojunction. a)** Output curve (*I*_ds_-*V*_ds_) of the Ta_2_NiSe_5_ FETs. Inset: optical image of the Ta_2_NiSe_5_ FETs, thickness measurement of the Ta_2_NiSe_5_ FETs. **b)** Output curve (*I*_ds_-*V*_ds_) of the SnS_2_ FETs at *V*_g_=15 V. Inset: optical image of the SnS_2_ FETs, thickness measurement of the SnS_2_ FETs. **c)** Output curves (*I*_ds_-*V*_ds_) of Ta_2_NiSe_5_/SnS_2_ heterojunction in dark with different gate voltage (*V*_g_).

**Figure S7.** Band diagram of the Ta_2_NiSe_5_/SnS_2_ heterojunction. **a)** before contact. **b)** After contact.

**Figure S8. a)** The time-resolved photoresponse of Ta_2_NiSe_5_/SnS_2_ device in air and in vacuum. **b)** The normalized of **a)**. The decay processes can be fitted well using the double exponential decay function:

$$I=I_{0}+C_{1}\text{exp}(-\frac{x-x_{0}}{\tau_{1}})+C_{2}\text{exp}(-\frac{x-x_{0}}{\tau_{2}})$$

where relaxation time constant *τ*_1_ and *τ*_2_ represent two relaxation processes with different rates: fast band-to-band transition (*τ*_1_) and slow carrier detrapping (*τ*_2_), of which the slow process with relaxation time constant *τ*_2_ plays the decisive role in determining the memory time. From figure, *τ*_2_, which determines the memory time, was boosted by almost an order of magnitude with the assistance of physisorption. **c)** The I-V curves at 532 nm of Ta_2_NiSe_5_/SnS_2_ device in air and in vacuum. It is noted that the photocurrent was almost unaffected by physisorption. All the measurements were performed at the same optical power.

**Figure S9. Output curves (I_ds_-V_ds_) of the Ta_2_NiSe_5_/SnS_2_ device under 375 nm to 1550 nm light illuminations.**

**Figure S10. Responsivity and photocurrent of the Ta_2_NiSe_5_/SnS_2_ device under 375 nm to 1550 nm light illuminations**

**Figure S11. Detectivity and EQE of the Ta_2_NiSe_5_/SnS_2_ device under 375 nm to 1550 nm light illuminations.**

**Figure S12. The synaptic behaviors of the Ta_2_NiSe_5_/SnS_2_ device in NIR. a)** EPSC. **b)** The dependence of the EPSC on the duration of light pulses. **c)** PPF. The dependence of the PPF index on the light pulses under **d)** 785 nm and **e)** 980 nm. **f)** STM-LTM.

**Supplementary Note 2. The relationship between EPSC peak value and incident light wavelength**

The reason that EPSC decreases with increasing wavelength is that the EPSC behavior is dominated by the photogating effect. As the wavelength increases, the photovoltage in the heterojunction decreases, resulting in a decrease in the peak value of the EPSC. The detailed proof is as follows.

As shown in Figure S13a, we observe in the experiment that the peak value of EPSC decreases with the increase of laser wavelength (*λ*). The EPSC behavior is caused by the photogating effect, which is mediated by photogenerated holes blocked by the interface barrier^1^. The typical characteristics of the EPSC behavior are: with the increase of illumination time, the photocurrent increases rapidly; after light illumination, the photocurrent decays slowly. As shown in Figure S13b, with light illumination, the photogenerated carriers generated by SnS_2_ are transported to the electrodes under the electric field. However, among photogenerated carriers generated by Ta_2_NiSe_5_, only photogenerated electrons can be transported, while photogenerated holes will be blocked by the interfacial potential barrier. The trapped photogenerated holes create a photogating effect, causing the electrons to undergo multiple cycles in the heterojunction channel, resulting in a significant photocurrent. The prolonged duration of illumination results in the accumulation of photogenerated holes, leading to a significant increase in photocurrent. As shown in Figure S13c, after light illumination, the blocked photogenerated holes will persist for a certain duration and continue to induce electron cycles until all the blocked photogenerated holes are recombined, resulting in a gradual decay of photocurrent. The blocked photogenerated holes play a crucial role in the manifestation of EPSC behavior.

To explore the relationship between the blocked photogenerated holes and the laser wavelength (*λ*), we measured the transfer curves (*I*_DS_-*V*_G_) of the Ta_2_NiSe_5_/SnS_2_ device, as shown in Figure S14. The threshold voltage shift (Δ*V*_th_) of transfer curves (I_DS_-V_G_) indicates the number of blocked photogenerated holes^2^. In Figure S14, the transfer curves shift to the left and exhibit a significant threshold voltage shift (Δ*V*_th_), which indicates the accumulation of photogenerated holes and the occurrence of the photogating effect. However, the threshold voltage shift (Δ*V*_th_) decreases from 28 V to 4.5 V as the laser wavelength (*λ*) increases from 405 nm to 980 nm, reflecting a reduction in accumulated photogenerated holes and a weakening of the photogating effect. Therefore, the peak value of EPSC decreases as the laser wavelength increases, which is consistent with the phenomenon in Figure S13a.

**Figure S13. a)** An EPSC of Ta_2_NiSe_5_/SnS_2_ device triggered by an optical pulse. The duration of the optical pulses is 200 ms. The power of the optical pulses from 375 nm to 1310 nm are 0.15 μW, 0.36 μW, 0.26 μW, 1.89 μW,5.66 μW, 21.05 μW, and 1.19 mW. **b)** Band diagrams of the junction under light illumination. **c)** Band diagrams of the junction after light illumination.

**Figure S14.** **Transfer curves (I_DS_-V_G_) in dark and under illumination with different wavelength of light.**

**Figure S15. a)** Multilevel states of Ta_2_NiSe_5_/SnS_2_ device under different light intensity and **b)** Zoomed-in view of the **a)** plot after 30 s.

**Figure S16.** The decay processes of **a)** STP and **b)** LTP can be well fitted with R^2^=0.99 by using the double exponential decay function.

**Figure S17. a)** EPSC curve and its dynamic range. **b)** LTP curve and corresponding its linearity fitting under 635 nm. The *V*_ds_ and power density of the optical pulses are -3 V and 2.3 μW.

**Supplementary Note 3. The impact of the gas environment on the synaptic performance of the Ta_2_NiSe_5_/SnS_2_ device.**

The surface of SnS_2_ contains a significant number of intrinsic sulfur vacancies, which serve as favorable adsorption sites for O_2_ molecules present in the surrounding air. The physisorption of O_2_ molecules can be elucidated by the following equation^3^:

$$\text{O}_{\text{2}}\left( \text{gas} \right)\overset{\Leftrightarrow}{\text{ }}\text{O}_{\text{2}}\left( \text{adsorption} \right)$$

$$\text{O}_{\text{2}}\left( \text{adsorption} \right)\text{+}\text{e}^{\text{-}}\overset{\Leftrightarrow}{\text{ }}\text{O}_{\text{2}}^{\text{-}}\text{(adsorption)}$$

The O_2_ molecules in the air combine with sulfur vacancies on the surface of SnS_2_, leading to the formation of surface adsorption, as shown in Figure S18. This surface adsorption has the capability to capture electrons within the channel, thereby inducing an abundant surface state. The presence of these surface states will significantly augment the capacity for charge storage and improve synaptic performance.

To validate the PAPPC effect in more depth, we investigate the impact of the gas environment on the synaptic performance of the Ta_2_NiSe_5_/SnS_2_ device. For all-in-one neuromorphic sensors, the real-time recognition/decoding of vision information is closely related to the paired-pulse facilitation (PPF) behavior. The *PPF index* is a measure of PPF performance and the *PPF index* is defined by the following equation:

$$PPF index=\left( {A_{1}}/{A_{2}} \right)\times100\%$$

We measured the PPF behavior of the Ta_2_NiSe_5_/SnS_2_ device in different atmospheres to evaluate the potential of the device in processing real-time visual recognition tasks, as shown in Figure S19. In Figure S19a, the PPF behavior of the device in a vacuum is not readily apparent, with a *PPF index* of only 109%, which can be attributed to the absence of oxygen molecules in the vacuum environment. We regulate the synaptic performance of the device by altering the oxygen concentration in the gaseous environment. In Figure S19b-d, the PPF behavior of the Ta_2_NiSe_5_/SnS_2_ device exhibits a more pronounced PPF behavior when the oxygen content in the gas environment is increased from 21% to 100%, resulting in an increase of the *PPF index* from 112% to 152%, which will lead to a better performance in processing real-time tasks. Then, we calculated the energy consumption of the device by using the following equation^4^:

where *V*_D_ is the drain-source voltage and *I*_D_ is the device current per spike with the duration of t and *n* is the number of optical spikes. In Figure S19e, the consumption of the device to complete a PPF behavior decreased from 710.42 pJ to 15.77 pJ (almost a 45-fold reduction) as the oxygen amount increased from 0% to 100%. The experimental results demonstrate that the PAPPC effect can enhance synaptic performance and significantly reduce power consumption. The power consumption can be further reduced by decreasing the bias voltage from 3 V to a much smaller value.

The transition from short-term plasticity (STP) to long-term plasticity (LTP) is essential for pattern recognition. The dynamic range (DR) indicates the potential to contain multiple conductance states and is crucial for high-precision neuromorphic computing. The dynamic range is defined by the following equation:

*dynamic range* = 20log(*PSC*_max_/*PSC*_min_)

where *PSC*_max_ is steady state PSC after 30_th_ pulse, and *PSC*_min_ is steady PSC after 1_st_ pulse. We measured the transition from STP to LTP in different gas environments to evaluate the potential of the device in processing high-precision neuromorphic computing, as shown in Figure S20. In Figure S20, the LTP of Ta_2_NiSe_5_/SnS_2_ device gradually improved as the oxygen amount ranged from 0 to 100%, resulting in an increase in DR from 5.54 dB to 9.46 dB and a 37-fold reduction in power consumption. The PAPPC effect holds significant potential for enhancing synaptic performance while simultaneously reducing power consumption.

The synaptic performance can be significantly enhanced by gas molecules, but it could be easily affected by environmental changes and become unstable. This problem can be effectively solved by encapsulating the synaptic transistors in a specific gas environment. In the future, the applicability of optoelectronic synaptic transistors could be extended to multimodal neuromorphic vision sensors for precise object recognition by employing the physisorption-assistant approaches. The fields rely on sensors to collect critical data about objects, including their color, odor, size, and other attributes. However, current sensor technology tends to rely on a single type of stimulus, and accuracy is often inadequate. In contrast, animals integrate a variety of sensory stimuli to improve recognition accuracy. For example, dogs use sight and smell to accurately recognize objects, such as tracking games or identifying their owners. Therefore, gas adsorption-assisted optoelectronic synaptic devices are expected to handle different types of stimuli and are becoming an important trend in sensing technology aiming at better recognition accuracy and robustness.

**Figure S18. Schematic illustrating the physisorption of O_2_ molecules in the air on the surface of the Ta_2_NiSe_5_/SnS_2_ device**.

**Figure S19. a-d)** The pair-pulse facilitation (PPF) behavior triggered by two consecutive optical pulses in different gas atmosphere at *V*_ds_ = -3 V. The duration time and interval time of the light pulses are equal, and the frequency of the light pulses is 15 Hz. To make the waveform undistorted, the sampling frequency of the oscilloscope is 50 Hz. Error bands represent the standard deviation obtained from four times independent tests. **e)** The impact of gas environment on the PPF index and power consumption.

**Figure S20. The transition from** **short-term plasticity (STP) to long-term plasticity (LTP) triggered by 30 consecutive pulses in different gas atmosphere at *V*_ds_ = -3 V**. The duration time and interval time of the light pulses are equal, and the frequency of the light pulses is 15 Hz. To make the waveform undistorted, the sampling frequency of the oscilloscope is 50 Hz. Error bands represent the standard deviation obtained from four times independent tests. **e)** The impact of gas environment on the DR and power consumption.

^^

**Figure S21. a)** Long-term potentiation (LTP) and Long-term depression (LTD) realized by 30 optical pulses and 30 gate voltage pulses. The wavelength, duration, interval, and power density of the optical pulses are 635 nm, 200 ms, 200 ms, and 13.26 μW. The duration, interval, and voltage of the gate voltage pulses are 200 ms, 200 ms, and 3 V. All measurements were performed at *V*_ds_ = -3 V. The energy band diagrams for the gate-tunable Long-term plasticity at **b)** after 30 optical pulses and **c)** under gate voltage pulses and **d)** after gate voltage pulses.

**Figure S22.** Imitation of classic Pavlovian conditioned reflex behavior under **a)** one and **b)** five trainings (E+L). The duration, interval, intensity of the optical pulses are 2 s, 2 s, and 218.99 μW. The duration, interval, and voltage of the gate voltage pulses are 2 s, 2 s, and 0.1 V.

**Table S1** Comparison of optoelectronic synapses by different materials.

| Materials | Wavelength(nm) | R(A/W) | power consumption(pJ) | PPF index | Year | Ref |
| --- | --- | --- | --- | --- | --- | --- |
| MoSe_2_+Bi_2_Se_3_+ PMMA/pentacene | 550-820 | - | 3000 | 115.66% | 2020 | [5] |
| ReS_2_ flake | 623 | - | 13 | - | 2020 | [6] |
| CdS/CdSe QDs/a-IGZO | 405-635 | 197.5(405 nm)  87.2(519 nm)  15(635 nm) | 13.03(405 nm)  78.33(519 nm)  0.5(635 nm) | 280%  (405 nm) | 2022 | [7] |
| In_2_Se_3_/MoS_2_ | 1060 | - | - | - | 2022 | [8] |
| PbS QDs/PMMA/pentacene | 365-850 | - | 0.00055 | 130%  (375 nm) | 2023 | [9] |
| Sn-MoO_3_ | 650-1550 | - | 40000 | 199.50%  (1570 nm) | 2023 | [10] |
| ReS_2_ | 450-650 | - | 0.01212 | 125% | 2023 | [11] |
| MoSe_2_ | 490-1060 | - | - | - | 2024 | [12] |
| CuInSe_2_ QDs/P3HT | 365-850 | - | 7500 | 130%(365 nm) 122%(500 nm) 115%(850 nm) | 2023 | [13] |
| Fe_7_S_8_@MoS_2_ | 365 | 10.2(365 nm) | - | - | 2024 | [14] |
| SWCNT TFTs | 365-620 | **-** | 3390 | 180%(620 nm) | 2024 | [15] |
| Ta_2_NiSe_5_/SnS_2_ | 375-1310 | 5610(405 nm)  14.4(980 nm) | 7.88 | 158%(635 nm) | 2024 | This work |

**References:**

1. Fang, H. et al. Photogating in Low Dimensional Photodetectors. *Advanced Science.* **4**, 1700323 (2017).

2. Island, J. O. et al Gate Controlled Photocurrent Generation Mechanisms in High-Gain In_2_Se_3_ Phototransistors. *Nano Letters.* **15**, 7853–7858 (2015).

3. Mishra, R. K. et al. Recent progress in gas sensing based on 2D SnS_2_ and its heterostructure platforms: A review. *Sensors and Actuators A: Physical.* **365**, 114860 (2024).

4. Yin, L. et al. Optically Stimulated Synaptic Devices Based on the Hybrid Structure of Silicon Nanomembrane and Perovskite. *Nano Letters.* **20**, 3378–3387 (2020).

5. Wang, Y. et al. Near‐Infrared‐Irradiation‐Mediated Synaptic Behavior from Tunable Charge‐Trapping Dynamics. *Advanced Electronic Materials.* **6**, 1900765 (2020).

6. John, R. A. et al. Optogenetics inspired transition metal dichalcogenide neuristors for in-memory deep recurrent neural networks. *Nature Communications.* **11**, 3211 (2020).

7. Jo, C. et al. Retina‐Inspired Color‐Cognitive Learning via Chromatically Controllable Mixed Quantum Dot Synaptic Transistor Arrays. *Advanced Materials.* **34**, 2108979 (2022).

8. Hu, Y. et al. Flexible Optical Synapses Based on In_2_Se_3_ /MoS_2_ Heterojunctions for Artificial Vision Systems in the Near-Infrared Range. *ACS Applied Materials & Interfaces* **14**, 55839–55849 (2022).

9. Zhang, J. et al*.* Retina‐Inspired Artificial Synapses with Ultraviolet to Near‐Infrared Broadband Responses for Energy‐Efficient Neuromorphic Visual Systems. *Advanced Functional Materials.* **33**, 2302885 (2023).

10. Kang, Y. et al*.* Bioinspired activation of silent synapses in layered materials for extensible neuromorphic computing. *Journal of Materiomics* **9**, 787–797 (2023).

11. Chen, Y. et al*.* Energy-Efficient ReS_2_ -Based Optoelectronic Synapse for 3D Object Reconstruction and Recognition. *ACS Applied Materials & Interfaces* **15**, 58631–58642 (2023).

12. Yang, H. et al*.* Near‐Infrared Optical Synapses Based on Multilayer MoSe_2_ Moiré Superlattice for Artificial Retina. *Advanced Functional Materials.* **34**, 2308149 (2024).

13. Zhang, J. et al*.* Energy‐efficient organic photoelectric synaptic transistors with environment‐friendly CuInSe_2_ quantum dots for broadband neuromorphic computing. *SmartMat* **5**, e1246 (2024).

14. Deng, Y. et al*.* Intrinsic Defect‐Driven Synergistic Synaptic Heterostructures for Gate‐Free Neuromorphic Phototransistors. *Advanced Materials.* 2309940 (2024).

15. Wang, Z. et al*.* Enhancement-Mode Carbon Nanotube Optoelectronic Synaptic Transistors with Large and Controllable Threshold Voltage Modulation Window for Broadband Flexible Vision Systems. *ACS Nano* **18**, 14298–14311 (2024).
